# Supplementary material for: Genome-wide maps of nucleolus interactions reveal distinct layers of repressive chromatin domains
Source: Nat Commun. 2022 Mar 18;13:1483. doi: 10.1038/s41467-022-29146-2 (PMC8933459; doi:10.1038/s41467-022-29146-2)
Supplement: Supplementary file 1 — Supplementary information [file 41467_2022_29146_MOESM1_ESM.pdf]

Supplementary information

Genome-wide maps of nucleolus interactions reveal distinct layers of repressive chromatin domains

Cristiana Bersaglieri, Jelena Kresoja-Rakic, Shivani Gupta, Dominik Bär, Rostyslav Kuzyakiv, Martina Panatta, Raffaella Santoro

Table of content

Supplementary Figures 1-10

Supplementary Tables 1-3

Supplementary References

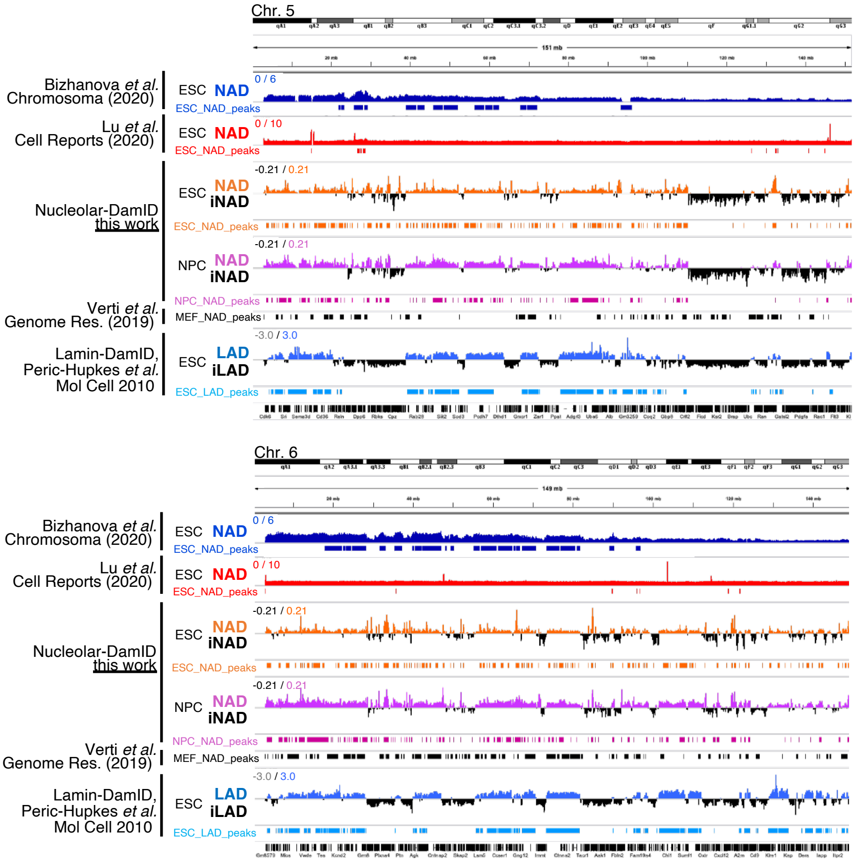

Supplementary Figure 1: Comparison of NAD profiles obtained with biochemically purified nucleoli and Nucleolar-DamID.

Chromosomal view of NADs obtained from purified nucleoli of mouse ESCs (Bizhanova et al., 2020; Lu et al., 2020) and MEFs (Vertii et al., 2019). NAD of ESCs and NPCs profiles obtained in this study with the Nucleolar-DamID and LADs of ESCs obtained with LaminB1-DamiD are shown (Peric-Hupkes et al., 2010),

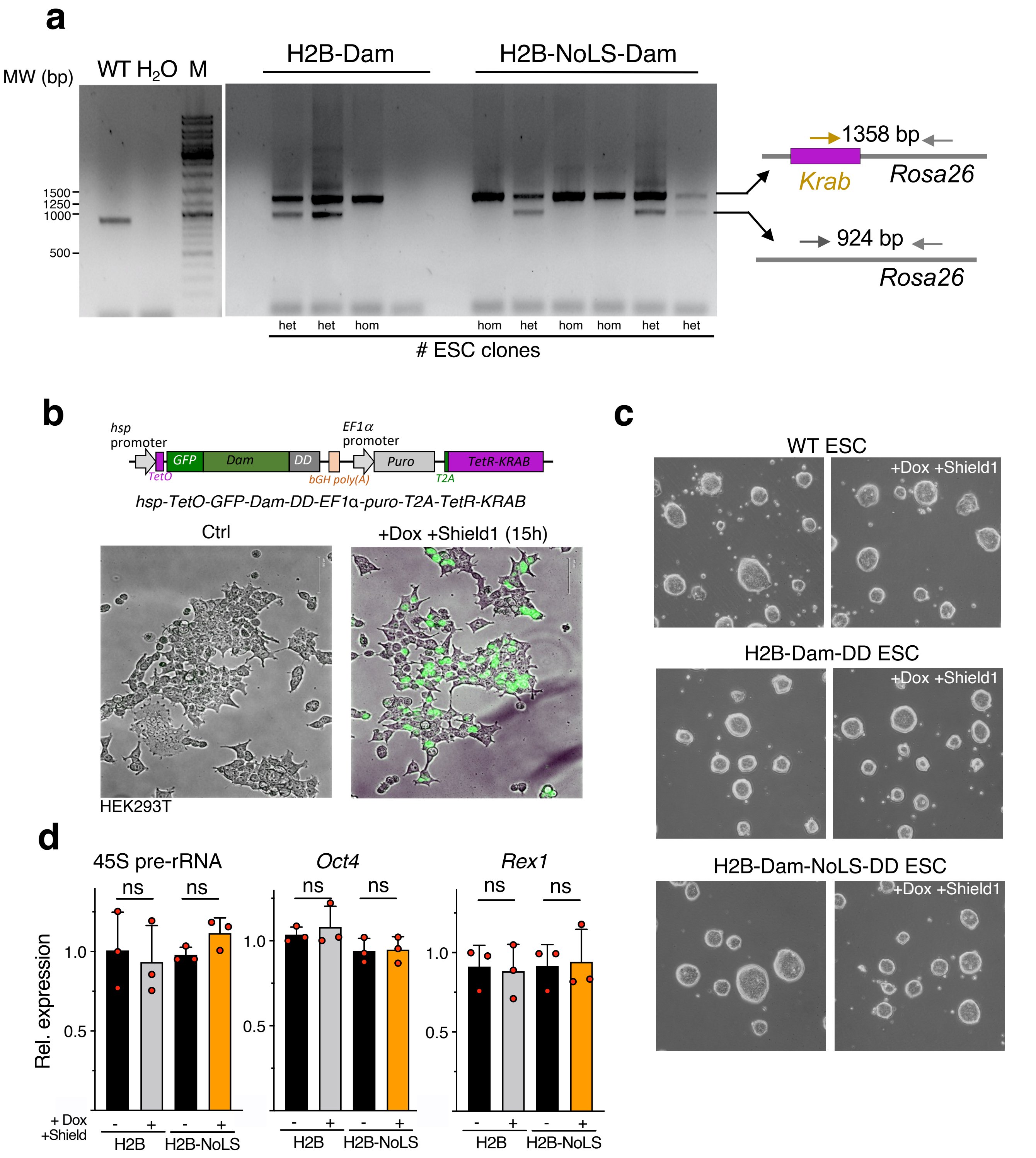

Supplementary Figure 2
Establishment of Nucleolar-DamID

a. PCR genotyping of ESC clones for the insertion of the H2B or nucleolar H2B sequences into *Rosa26* locus. het.: heterozygotic insertion; hom.: homozygotic insertion; WT: wild type ESCs; M: DNA marker. **b.** Life cell images showing GFP signal in HEK293T transfected with the *hsp-TetO-GFP-Dam-DD-EF1*α*-puro-T2A-TetR-KRAB* plasmid and treated without or with 1 µg/ml doxycycline (Dox) and 1 µM Shield1 for 15 hours. **c.** Representative bright field images of ESC lines expressing H2B-Dam-DD and H2B-Dam-NoLS-DD transgenes and parental ESCs (WT) without and after 15 hours treatment with 100 ng Doxycycline (Dox) and 1 µM Shield1. **d.** qRT-PCR showing similar expression levels of 45S pre-rRNA and the pluripotency genes *Oct4* and *Rex1*. Mean values from data of three biologically independent experiments. Error bars represent s.d.. Statistical significance (*P*-values) was calculated using the paired two-tailed t-test. ns: non-significant. Source data are provided as a Source Data file.

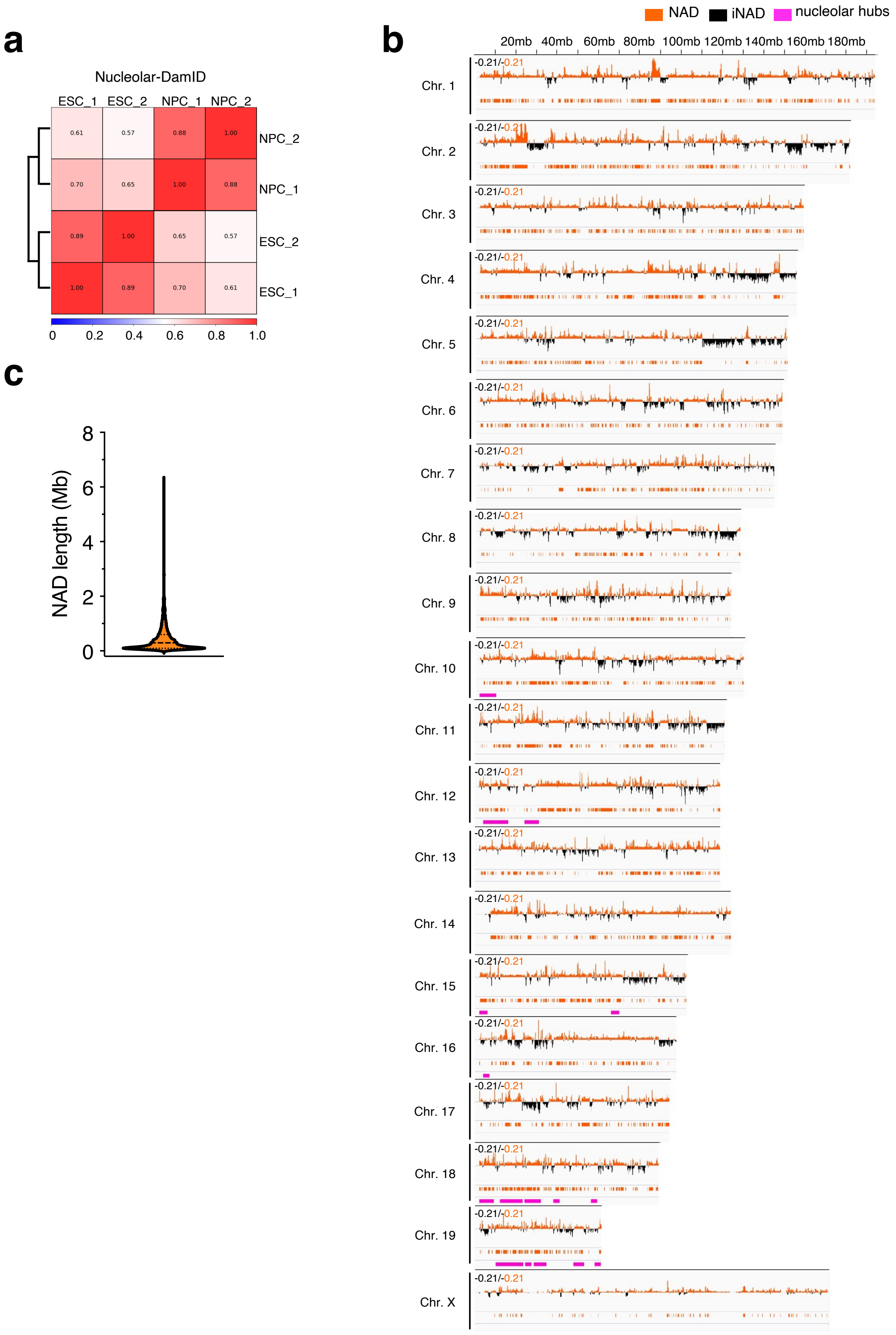

Supplementary Figure 3
Nucleolar-DamID in ESCs

a. Pearson correlation of Nucleolar-DamID experiments in ESCs and NPCs. **b.** Chromosomal view of NADs in ESCs. NADs are measured as log_2 ratio of m6A levels between H2B-Dam-NoLS vs. H2B-Dam. iNAD: inter-NAD regions. iLAD: inter-LAD regions. Orange bars correspond to significant NADs. Pink bars correspond to sequence found in nucleolar hubs using the SPRITE method [34]. **c.** Violin plot showing distribution of NAD length. Source data are provided as a Source Data file.

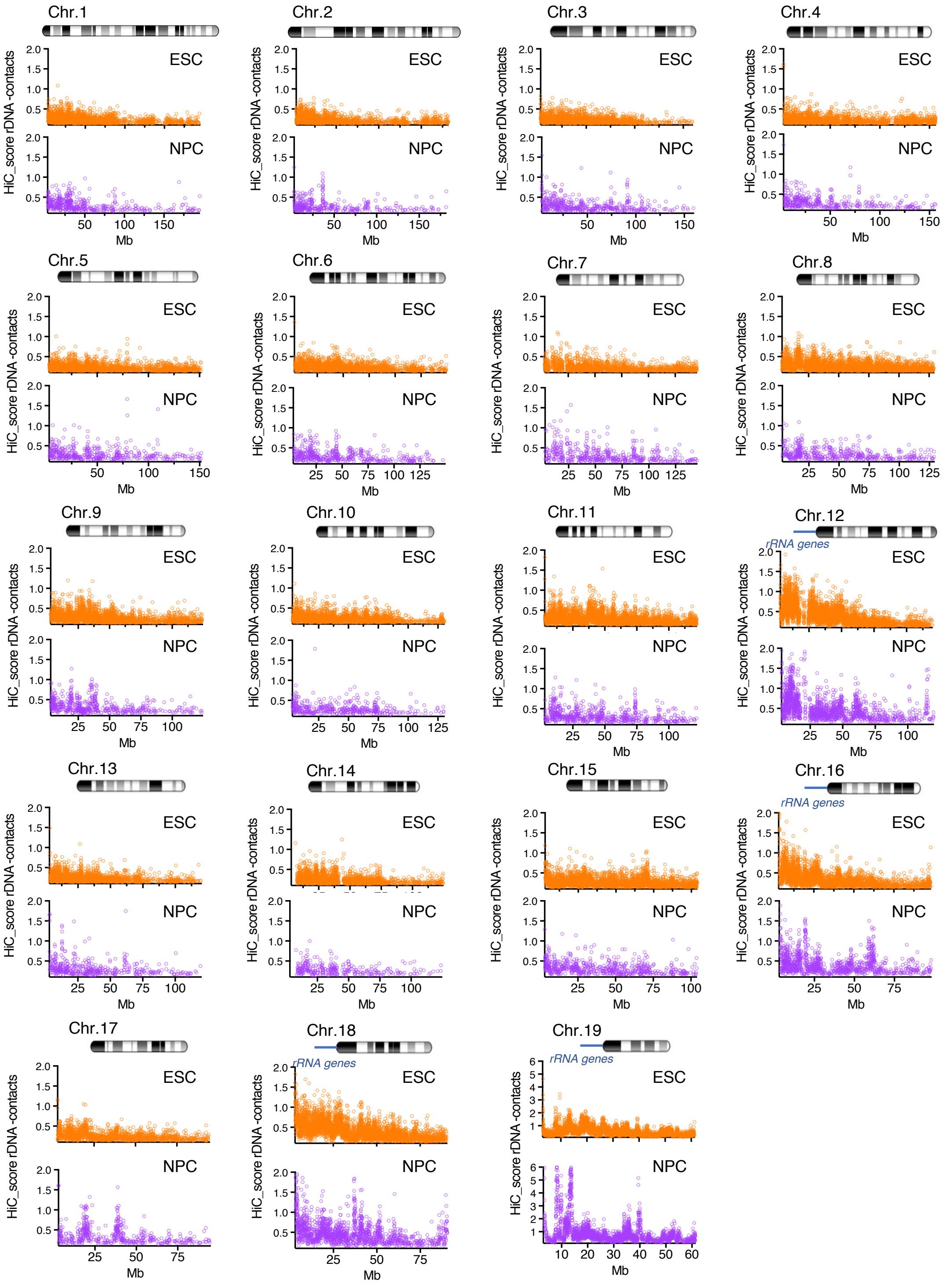

Supplementary Figure 4

rDNA contacts identified by HiC-rDNA in ESCs and NPCs

Count score of rDNA contacts obtained by HiC-rDNA on chromosomes of ESCs (orange) and NPCs (magenta). Chromosomes containing rRNA genes (12, 16, 18, and 19) are indicated. Source data are provided as a Source Data file.

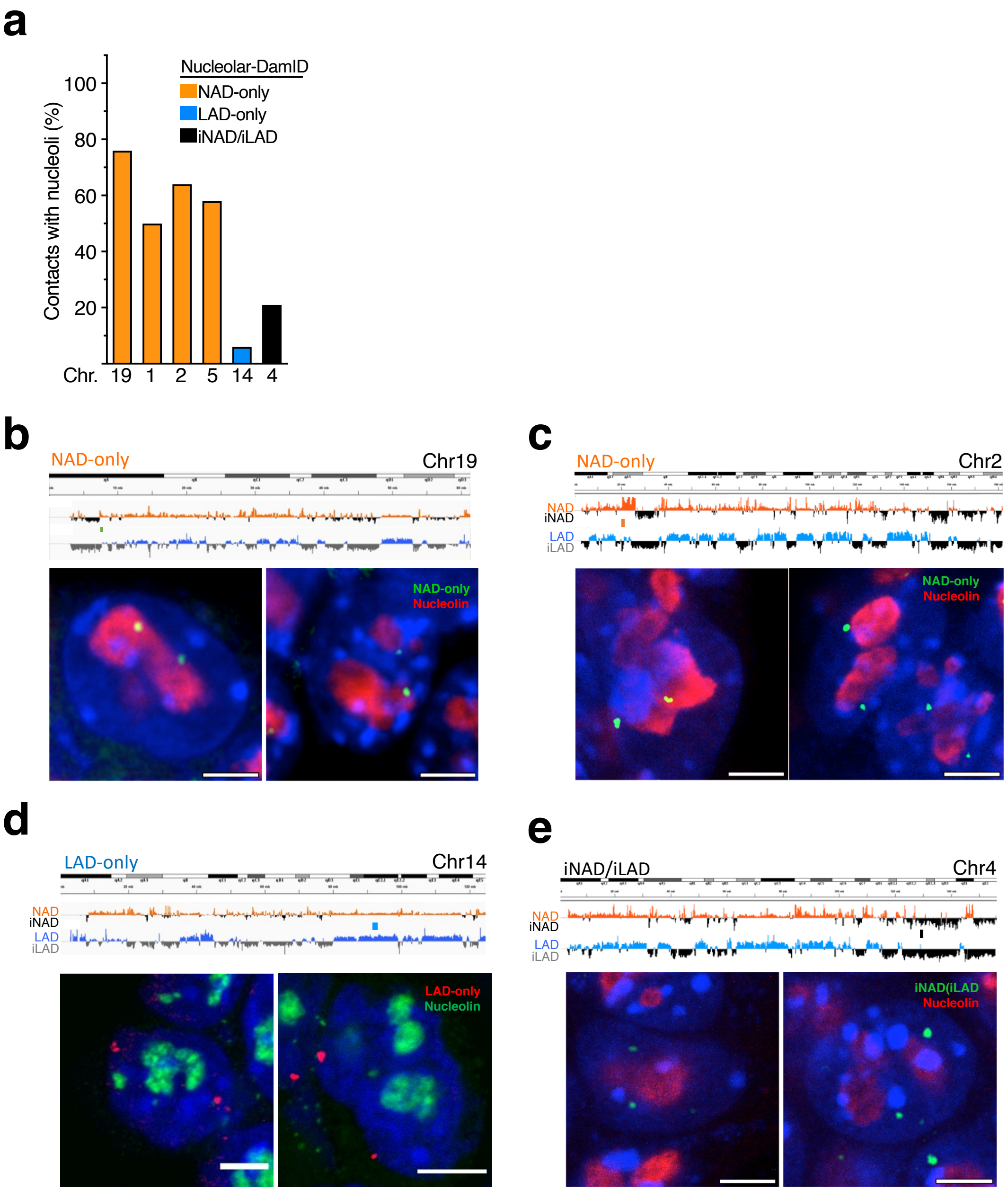

Supplementary Figure 5

NADs identified by Nucleolar-DamID contact the nucleoli of ESCs

a. Quantification of the contacts with the nucleolus (%) measured by DNA-FISH using the indicated probes. Data are from the measurements of 70-120 cells for each condition.

b-e. Upper panels represent NAD and LAD profiles of chromosome 19 (**b**), 2 (**c**), 14 (**d**), and 4 (**e**) and the DNA-FISH probes hybridizing to a region identified as NAD-only (orange bar), LAD-only (blue bar), or iNAD/iLAD (black bar). Lower panels. Example images from immunofluorescences for nucleolin combined with the corresponding DNA-FISH probe and DAPI (blue). Size bar is 5μm.

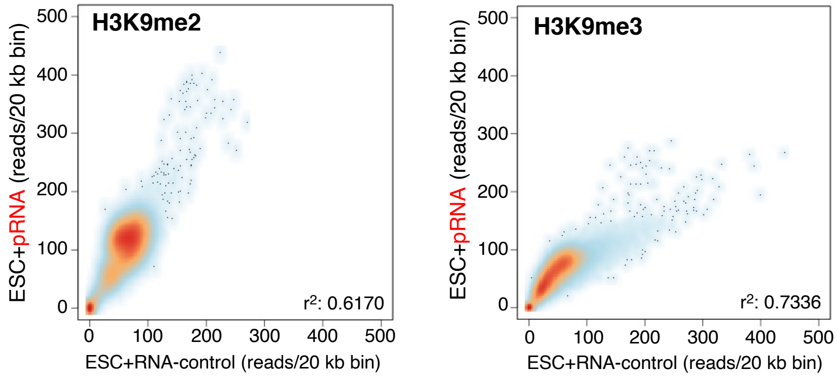

Supplementary Figure 6

pRNA-mediated heterochromatin formation at rRNA genes increases H3K9me2 at sequences adjacent to the nucleolus

a. Addition of pRNA in ESCs causes an increase in H3K9me2 at several genomic regions. Independent ChIPseq experiment of ESCs transfected with pRNA and RNA control. Scatter plot showing H3K9me2 and H3K9me3 levels (reads/20kb bin) between ESC+pRNA and ESC+RNA-control. Source data are provided as a Source Data file.

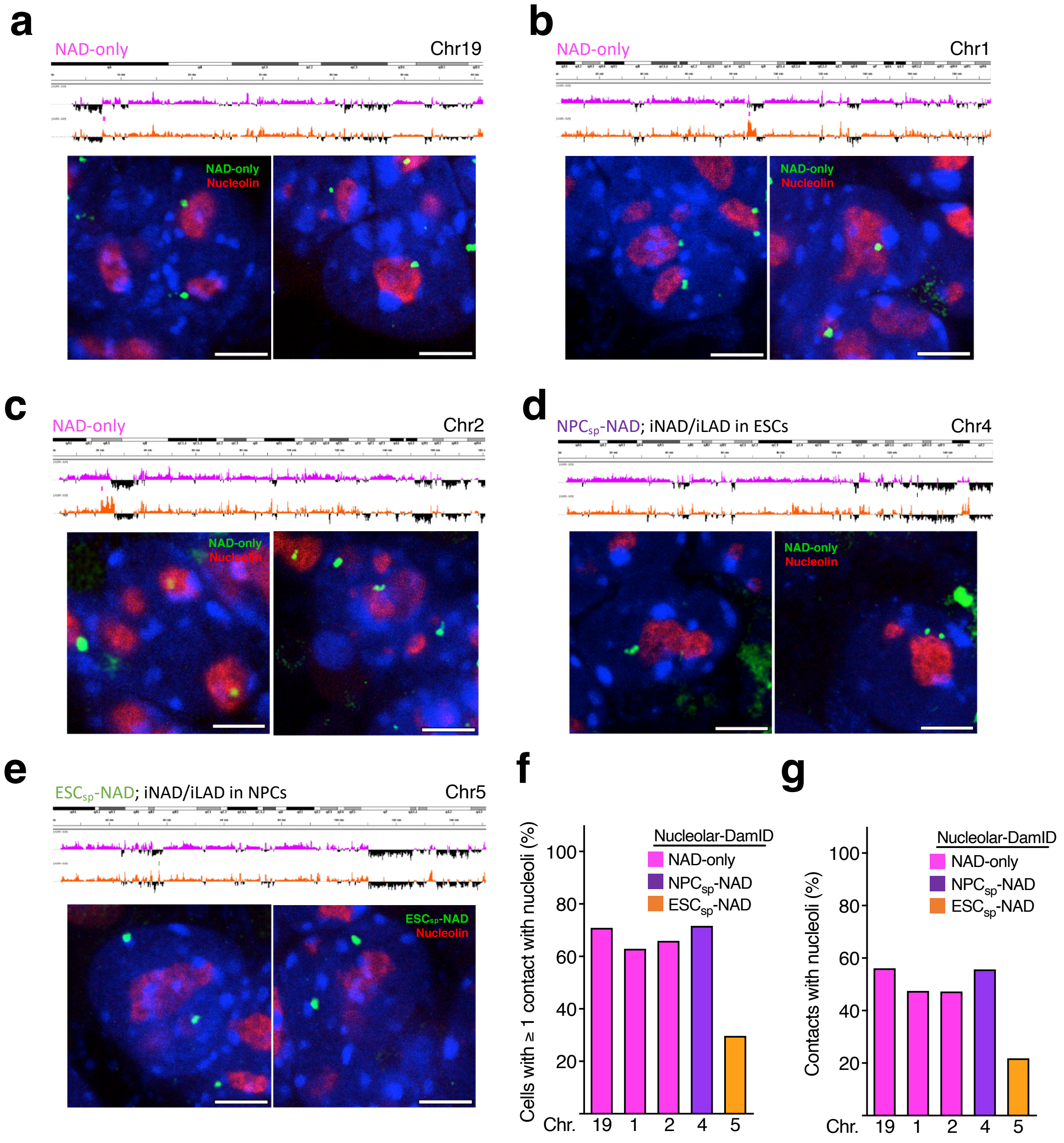

Supplementary Figure 7

NADs identified by Nucleolar-DamID contact the nucleoli of NPCs

a-e. Upper panels represent NAD profiles of ESCs and NPCs of chromosomes 19 (**a**), 1 (**b**), 2 (**c**), 4 (**d**), and 5 (**e**) and the DNA-FISH probes hybridizing to a region identified as NAD-only in both ESCs and NPCs (**a-c**, pink bar), NPC_{sp}-NAD (**d**, violet bar), or ESC_{sp}-NAD (**e**, orange bar). Lower panels. Example images from immunofluorescences for nucleolin (red) combined with the corresponding DNA-FISH probe (green) and DAPI (blue). Size bar is 5μm. **f.** Quantification of NPCs displaying at least one DNA-FISH probe signal that contacts the nucleolus. Data are from the measurements of 85 cells for Chr. 19 (NAD-only), 77 for Chr. 1 (NAD-only), 52 for Chr. 2 (NAD-only), 189 for Chr. 4 (NPC_{sp}-NAD) and 59 for Chr. 5 (ESC_{sp}-NAD). **g.** Quantification of contacts with the nucleolus measured by DNA-FISH using the indicated probes. Data are from the measurements of 128 DNA-FISH signals for Chr. 19 (NAD-only), 128 for Chr. 1 (NAD-only), 88 for Chr. 2 (NAD-only), 326 for Chr. 4 (NPC_{sp}-NAD) and 84 for Chr. 5 (ESC_{sp}-NAD).

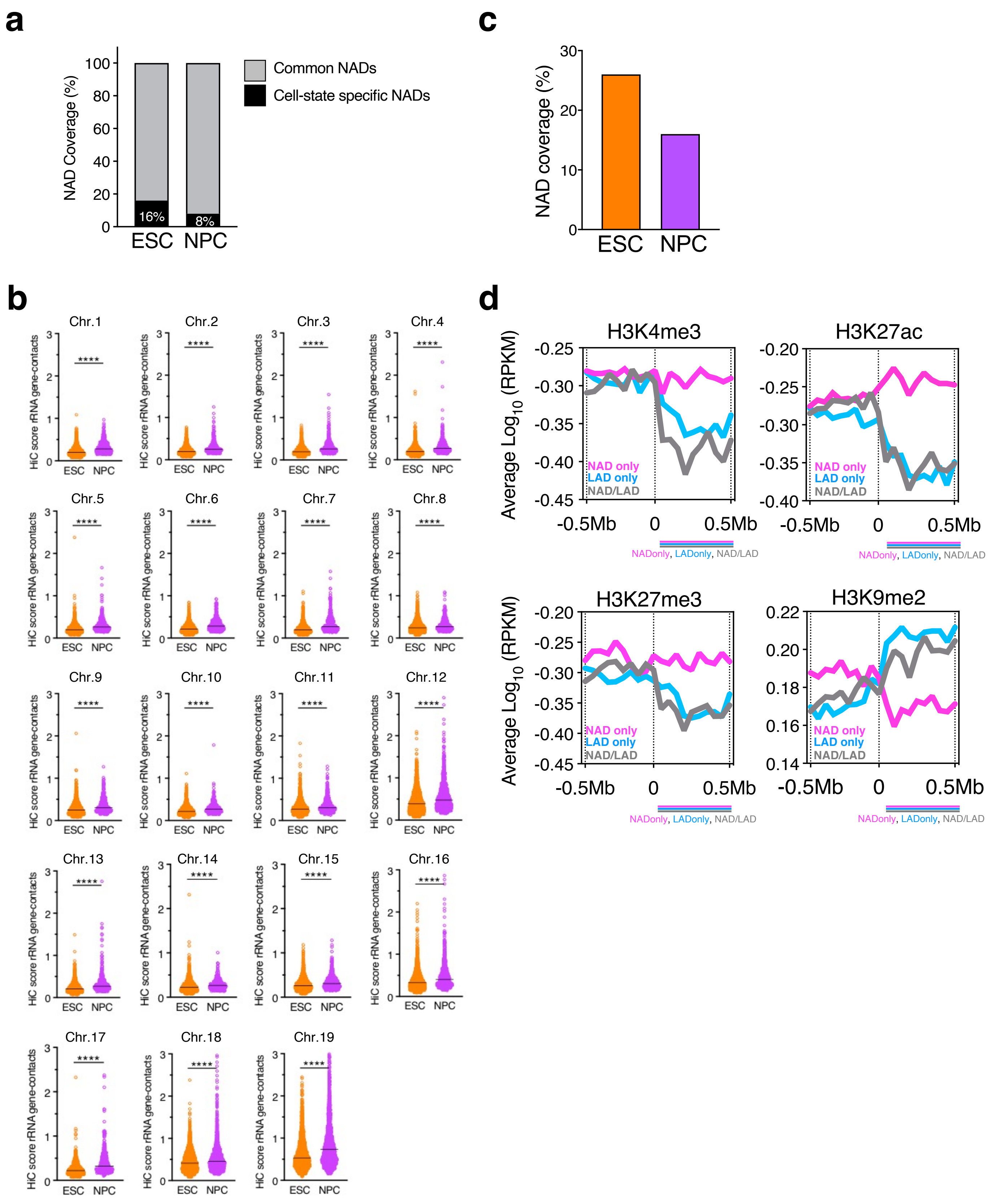

Supplementary Figure 8
rDNA contacts in ESCs and NPCs

a. % of NAD sequences common to ESC and NPC. **b.** Count score of rDNA contacts at each chromosome in ESCs (orange) and NPCs (magenta). Statistical significance (*P*-values) between ESCs and NPCs was calculated using the paired two-tailed t-test ****< 0.0001). **c.** NAD coverage in ESC and NPC. **d.** Occupancy (average RPKM) of histone modifications plotted over the boundaries of NAD-only, LAD-only, and NAD/LAD in NPCs, respectively. Source data are provided as a Source Data file.

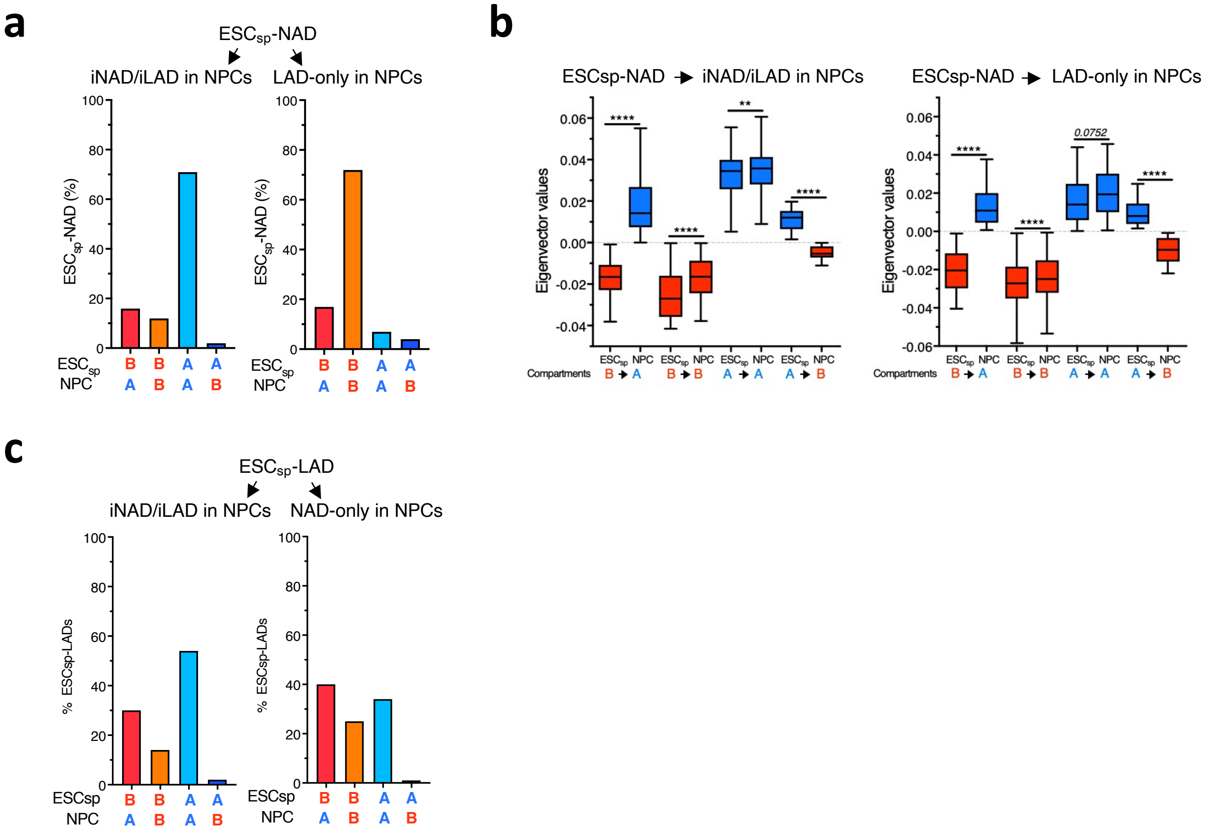

Supplementary Figure 9

Compartment features of cell type specific NADs

a. Values represent the proportion (%) of ESC_{sp}-NAD and their corresponding location in A and B compartments of ESCs and NPCs classified according to regions that move away from the nucleolus and nuclear lamina (iNAD/iLAD) or gain exclusive location at the nuclear periphery (LAD-only) in NPCs. **b.** Box plots showing eigenvectors values of ESC_{sp}-NADs in the active A and repressive B compartments. Statistical significance (*P*-values) was calculated using the paired two-tailed t-test (**< 0.01, ****< 0.0001). Box plots depict the minimum and maximum values. The mean is represented by a horizontal line within the boxes. **c.** Values represent the proportion (%) of ESC_{sp}-LAD and their corresponding location in A and B compartments of ESCs and NPCs classified according to regions that move away from the nucleolus and nuclear lamina (iNAD/iLAD) or gain exclusive location at the nuclear periphery (LAD-only) in ESCs. Source data are provided as a Source Data file.

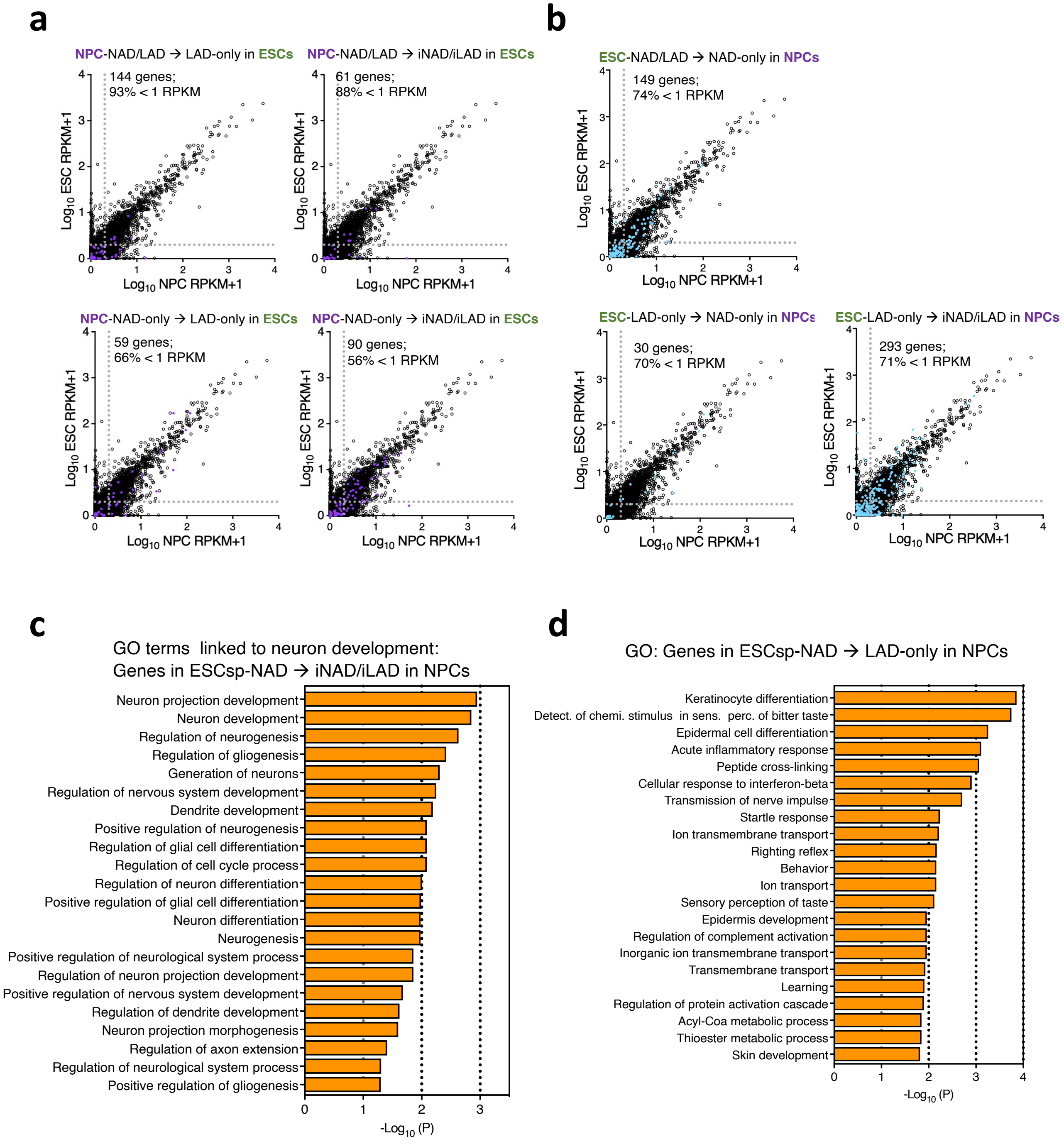

Supplementary Figure 10
Gene expression of cell type specific NADs

a. Scatter plot showing gene expression levels between ESC and NPCs. Expression of genes located at NPC_{sp}-NAD types (NAD/LAD and NAD-only) that became LAD-only or iNAD/iLAD in ESCs are highlighted in magenta whereas total genes are represented in black. Dotted lines indicate RPKM value as 1. **b.** Scatter plot showing gene expression levels between ESC and NPCs. Expression of genes located at ESC_{sp}-LAD types (NAD/LAD and LAD-only) that became NAD-only or iNAD/iLAD in NPCs are highlighted in blue whereas total genes are represented in black. Dotted lines indicate RPKM value as 1. **c.** Gene ontology terms of genes located at ESC_{sp}-NAD that become iNAD/iLAD in NPCs. **d.** Gene ontology terms of genes located at ESC_{sp}-NAD that became LAD-only in NPCs. Source data are provided as a Source Data file.

Primers

**Dam transgenes (qRT-PCR)**			
ORS1159 Dam	Forw.	TTCCCGAAACAAATTGCGCC	
ORS1160 Dam	Rev.	CCGAACGGCACGTTAAACTC	
ORS150_beta Actin	Forw.	CATCCAGGCTGTGCTGTCCCTGTATGC	
ORS151_beta Actin	Rev.	GATCTTCATGGTGCTAGGAGCCAGAGC	
**Dpn II digestion**			
rDNA -2272/2252	Forw.	ACCCTCCGGAGAGACAGAATG	
rDNA -2006/2026	Rev.	CCCTGTACGACCTCCTTGTTA	
rDNA +1/+20	Forw.	ACTGACACGCTGTCCTTTCC	
rDNA +111/+130	Rev.	GACAGCTTCAGGCACCGCGA	
ORS1370 Tuba1a	Forw.	CTTAAAGCATGGAGACCACCC	
ORS1371 Tuba1a	Rev.	CAGAGCTGTATATAAGGAGCCGC	
ORS890 Cdx2	Forw.	GTAGCCATTCCAGTCCTCGC	
ORS891 Cdx2	Rev.	TACCCGGACTACGGTGGTTA	
**45S pre-rRNA (qRT-PCR)**			
45S pre-rRNA +550/570	Forw.	CTCTTGTTCTGTGTCTGCC	Savic et al., Cell Stem Cell (2014)
45S pre-rRNA +745/765	Rev.	GCCCGCTGGCAGAACGAGAAG	
**Oct4 (qRT-PCR)**			
ORS521 Oct4	Forw.	GGCGTTCGCTTTGGAAAGGTGTTC	This work
ORS522 Oct4	Rev.	CTCGAACCACATCCTTCTCT	
**Rex1 (qRT-PCR)**			
ORS401 Rex1	Forw.	AGAAAGCAGGATCGCCTCAC	This work
ORS402 Rex1	Rev.	AGGGAACTCGCTTCCAGAAC	
**rRNA genes (ChIP)**			
rDNA promoter -165/-145	Forw.	GACCAGTTGTTCCTTTGAGG	Savic et al., Cell Stem Cell (2014)
rDNA promoter -21/-1	Rev.	ACCTATCTCCAGGTCCAATAG	
**Minor satellites (ChIP)**			
Minor Satellite	Forw.	CATGGAAAATGATAAAAACC	Martens et al., EMBO J. (2005)
Minor Satellite	Rev.	CATCTAATATGTTCTACAGTGTGG	
**Libraries DamID**			
**Adaptor DamID**			
AdRt		CTAATACGACTCACTATAGGGCAGCGTGGTCGCGGCCGAGGA	Vogel et al., Nature protocols (2007)
AdRb		TCCTCGGCCG	
**PCR amplification**			
AdR PCR		GGTCGCGGCCGAGGATC	Vogel et al., Nature protocols (2007)

Supplementary Table 1: List of primers used in this study

**FISH**	**Amount**			
Nucleolin	Dilution 1:500	rabbit polyclonal	Abcam	Cat# ab22758
**IF**				
NPM1	Dilution 1:500	mouse monoclonal	Sigma	Cat# B0556
Fibrillarin	Dilution 1:500	rabbit monoclonal	CellSignaling	Cat# 2639
**Secondary antibodies**				
IgG (H+L) Highly Cross-Adsorbed. Alexa Fluor 488	Dilution 1:500	rabbit polyclonal	ThermoFischer	Cat# A11034
IgG (H+L) Highly Cross-Adsorbed. Alexa Fluor 546	Dilution 1:500	rabbit polyclonal	ThermoFischer	Cat# A11035
**ChIPseq**				
H3K9me2	2ug Ab/20ug chromatin	mouse monoclonal	Abcam	Cat# ab1220
H3K9me3	2ug Ab/20ug chromatin	rabbit polyclonal	Abcam	Cat# ab8898

Supplementary Table 2: List of antibodies used in this study

FISH probes			
	**Chromosome**	**start**	**end**
**NAD**	chr19	7400268	7799149
**LAD**	chr14	91511514	92294979
**NAD**	chr1	86599041	87100810
**NAD**	chr2	20549286	21216571
**ESCsp-NAD**	chr5	37199822	37599742
**NPCsp-NAD**	chr4	129241670	129443207

DNA FISH OligoPaint libraries		
**Emulsion PCR**		
ORS2149 univ	Forward	GCGTTAGGGTGCTTACGTCTG
ORS2150 univ	Reverse	CACCTCCGTCTCTCACCTCTC
**PCR1**		
ORS2151 chr14/chr1	Forward	CACCGACGTCGCATAGAACGGAAGAGCGTGTGAGAGCGCG GTTGGATTAATGA
ORS2152 chr14/chr1	Reverse	P-CGGCTCATATCGGGAGACAAC
ORS2153 chr19/chr5	Forward	CACCGACGTCGCATAGAACGGAAGAGCGTGTGGGCTCTGAT CTCTGGGTACCT
ORS2154 chr19/chr5	Reverse	P-CTACGATAGCTTTGGCGCTCT
ORS2155 chr2/chr4	Forward	CACCGACGTCGCATAGAACGGAAGAGCGTGTGTGAATAGCA GCGGTGGCAAAC
ORS2156 chr2/chr4	Reverse	P-GCGTATGAGGAGGCGAGAGTA
**PCR2**		
ORS2157 Green univ	Forward	/5Alex488N/CACCGACGTCGCATAGAACGG

Supplementary Table 3: Description of DNA-FISH probes and primers used to obtain DNA-FISH oligopaint libraries

Supplementary references

Bizhanova, A., Yan, A., Yu, J., Zhu, L.J., and Kaufman, P.D. (2020b). Distinct features of nucleolus associated domains in mouse embryonic stem cells. Chromosoma 129, 121-139.

Lu, J.Y., Shao, W., Chang, L., Yin, Y., Li, T., Zhang, H., Hong, Y., Percharde, M., Guo, L., Wu, Z., et al.(2020). Genomic Repeats Categorize Genes with Distinct Functions for Orchestrated Regulation. Cell Rep 30, 3296-3311.e3295.

Peric-Hupkes, D., Meuleman, W., Pagie, L., Bruggeman, S.W., Solovei, I., Brugman, W., Graf, S., Flicek, P., Kerkhoven, R.M., van Lohuizen, M., et al. (2010). Molecular maps of the reorganization of genome nuclear lamina interactions during differentiation. Mol Cell 38, 603-613.

Quinodoz, S.A., Ollikainen, N., Tabak, B., Palla, A., Schmidt, J.M., Detmar, E., Lai, M.M., Shishkin, A.A.,Bhat, P., Takei, Y., et al. (2018). Higher-Order Inter-chromosomal Hubs Shape 3D Genome Organization in the Nucleus. Cell 174, 744-757 e724.

Vertii, A., Ou, J., Yu, J., Yan, A., Pages, H., Liu, H., Zhu, L.J., and Kaufman, P.D. (2019). Two contrasting classes of nucleolus-associated domains in mouse fibroblast heterochromatin. Genome Res 29, 1235-1249.
