# Supplementary material for: Genome-wide maps of nucleolus interactions reveal distinct layers of repressive chromatin domains
Source: Nat Commun. 2022 Mar 18;13:1483. doi: 10.1038/s41467-022-29146-2 (PMC8933459; doi:10.1038/s41467-022-29146-2)
Supplement: Supplementary file 3 — Description of additional Supplementary File [file 41467_2022_29146_MOESM3_ESM.pdf]

Descriptions of Additional Supplementary data Files

Supplementary Data 1 List of NADs in ESCs identified by NucleolarDamID.

Supplementary Data 2 List of rDNA contacts in ESCs and NPCs identified by HiCrDNA.

Supplementary Data 3 List of genes and gene ontology terms of NADs in ESCs

Supplementary Data 4 List of NADs in NPCs identified by NucleolarDamID. Supplementary Data 5 List of ESCsp- and NPCsprDNA contacts.

Supplementary Data 6 List of ESCsp- and NPCspNADs.

Supplementary Data 7 RNAseq of ESCs and NPCs

Supplementary Data 8 List of genes located at ESCsp- and NPCsp-rDNA contacts

Supplementary Data 9 Gene ontology of genes located at ESCsp- and NPCsprDNA contacts

Supplementary Data 10 List of genes located at ESCsp- and NPCsp-NADs Supplementary Data 11 Gene ontology of genes located at ESCsp- and NPCspNAD

Supplementary Data 11 Gene ontology of genes located at ESCsp- and NPCspNAD
