# Supplementary material for: Genome-wide maps of nucleolus interactions reveal distinct layers of repressive chromatin domains
Source: Nat Commun. 2022 Mar 18;13:1483. doi: 10.1038/s41467-022-29146-2 (PMC8933459; doi:10.1038/s41467-022-29146-2)
Supplement: Supplementary file 15 — Reporting Summary [file 41467_2022_29146_MOESM15_ESM.pdf]

nature research

Corresponding author(s): Raffaella Santoro

Last updated by author(s): 2022.02.01

Reporting Summary

Nature Research wishes to improve the reproducibility of the work that we publish. This form provides structure for consistency and transparency in reporting. For further information on Nature Research policies, see our Editorial Policies and the Editorial Policy Checklist.

Statistics

For all statistical analyses, confirm that the following items are present in the figure legend, table legend, main text, or Methods section.

n/a	Confirmed	
☐	☒	The exact sample size (*n*) for each experimental group/condition, given as a discrete number and unit of measurement
☐	☒	A statement on whether measurements were taken from distinct samples or whether the same sample was measured repeatedly
☐	☒	The statistical test(s) used AND whether they are one- or two-sided<br>*Only common tests should be described solely by name; describe more complex techniques in the Methods section.*
☒	☐	A description of all covariates tested
☒	☐	A description of any assumptions or corrections, such as tests of normality and adjustment for multiple comparisons
☐	☒	A full description of the statistical parameters including central tendency (e.g. means) or other basic estimates (e.g. regression coefficient) AND variation (e.g. standard deviation) or associated estimates of uncertainty (e.g. confidence intervals)
☐	☒	For null hypothesis testing, the test statistic (e.g. *F*, *t*, *r*) with confidence intervals, effect sizes, degrees of freedom and *P* value noted<br>*Give P values as exact values whenever suitable.*
☒	☐	For Bayesian analysis, information on the choice of priors and Markov chain Monte Carlo settings
☒	☐	For hierarchical and complex designs, identification of the appropriate level for tests and full reporting of outcomes
☒	☐	Estimates of effect sizes (e.g. Cohen's *d*, Pearson's *r*), indicating how they were calculated

Our web collection on statistics for biologists contains articles on many of the points above.

Software and code

Policy information about availability of computer code

Data collection

ChIPseq was performed on the Illumina HiSeq 2500 using single end sequencing. Nucleolar-DamID libraries were sequenced on the Novaseq 6000 using single end reads. RNAseq was performed on the Novaseq 6000 using single end sequencing.

Data analysis

Nucleolar-DamID and ChIPseq Fastaq files were aligned to the mm10 reference genome assembly (UCSC) using Bowtie2 (version 2.3.4.3) with default parameters. Resulting sam files were converted into bam files, sorted and indexed using samtools (version 1.9).
For Nucleolar-DamID, the bam files were analyzed using the damidseq pipeline script from the Brand group (http://owenjm.github.io/damidseq_pipeline) (Marshall and Brand, 2015, PMID: 26112292) at 100kbp resolution. The pipeline gives a bedgraph file with the log2 ratio of the m6A between H2B-Dam-NoLS and the H2B-Dam only, visualized using the tool Integrative Genome Viewer (IGV, version 2.5.2). The bedgraph files were converted into bigwig files using the bedGraphToBigWig UCSC package (version 4) and the Pearson correlation was assessed using "multiBigwigSummary" and "plotPCA" from deepTools (version 3.2.1). The bedgraph files were processed with the find_peaks software associated with the pipeline (https://github.com/owenjm/find_peaks) adjusting the values of the FDR and the minimum quantile (FDR <0.01 and min_quant 0.70). Only the significative peaks common to both replicates were considered as NADs for further analysis. The identification of NADs overlapping with LADs, genomic contacts with rRNA genes identified by HiC-rDNA, A and B compartment, early/late replicating regions, and ESCsp- and NPCsp-NADs, ESCsp- and NPCsp-LADs were performed using "Intersect intervals" from bedtools (version 2.28.0). NAD-only and NAD/LAD distribution over the chromosomes, was generated with ChIPseeker (Yu et al., 2015, PMID: 25765347) in R studio (version 1.0.44). GO term analysis was performed using DAVID 6.8 (Huang et al., 2009, PMID: 19131956).
For ChIP seq data,read counts were computed and normalized using "bamCoverage" from deepTools (version 3.2.1) using a bin size of 50bp. To calculate read coverage for 20kb bin region of H3K9me2 and H3K9me3 ChIPseq, "multiBamSummary" from deepTools (version 3.2.1) was used. The border profiles and the read coverage box plots were generated using deepTools (version 3.2.1). H3K9me2 increase in ESC+pRNA regions distribution over the chromosomes was generated with the ChIPseeker package (Yu et al., 2015, PMID: 25765347). Integrative Genome Viewer (IGV, version 2.5.2) was used to visualize and extract representative ChIPseq tracks.
For HiC-rDNA, the identification of genomic contacts with rRNA genes was performed by recovering reads containing rRNA gene contacts from three published Hi-C data of ESCs and NPCs (Bonev et al., 2017, PMID: 29053968). The obtained Hi-C data sets have been analyzed with Juicer (Durand et al., 2016, PMID: 27467249) all in one computational pipeline for generating Hi-C maps from raw fastq data files and

command line tools for feature annotation on the Hi-C maps. During Juicer analysis, raw fastq data sets have been aligned to the customized mm10 reference genome with Burrows-Wheeler Aligner (Li and Durbin, 2009, PMID: 19451168) under default parameters. The modified mm10 genome contained one rRNA gene unit attached to the end of chromosome 12. The chromosomal interaction have been extracted from interaction matrices (hic files) with Juicebox tools command dump under the following parameters (contacts: observed, normalization applied: Knight-Ruiz matrix balancing (Knight and Ruiz, 2012) under base-pair delimited resolution with bin size 5000). ENCODE Data Analysis Consortium Blacklisted Regions (Hoffman et al., 2013, PMID: 23221638) were excluded from the analysis with bedtools (version 2.28.0). Only Hi-C reads contacting rRNA gene sequences and other genomic sequences have been selected for further analysis through the computation with bedtools pairtoBED function (version 2.28.0) and Python Pandas Library (https://pandas.pydata.org/). Common contacts between the three Hi-C replicates were identified using HiCcompare (Stansfield et al., 2018, PMID: 30064362), running the tool under default parameters.
For RNA seq, the quality of the 120 bp single end reads generated by the machine was checked by FastQC, a quality control tool for high throughput sequence data. The quality of the reads was increased by applying: a) SortMeRNA (Kopylova, 2012, PMID: 23071270) (version 2.1) tool to filter ribosomal RNA; b) Trimmomatic (Bolger, 2014, PMID: 24695404) (version 0.36) software package to trim the sorted (a) reads. The sorted (a), trimmed (b) reads were mapped against the mouse genome (mm10) using the default parameters of the STAR (Spliced Transcripts Alignment to a Reference, version 2.4.0.1) (Dobin et al., 2013, PMID: 23104886). For each gene, exon coverage was calculated using a custom pipeline and then normalized in reads per kilobase per million (RPKM) (Mortazavi et al., 2008, PMID: 18516045), the method of quantifying gene expression from RNA sequencing data by normalizing for total read length and the number of sequencing reads.
DNA-FISH probes for chromosomes 1, 2, 4, 5, and 19 were generated with oligopaint libraries that were constructed the PaintSHOP interface created by the Beliveau lab (https://oligo.shinyapps.io/paintshop/_w_33571817/#tab-1201-8) (Hershberg et al., 2021, PMID: 34226720). Each library contains a universal primer pair used to amplify all the probes in the library, followed by a specific primer pair hooked to the 40-46-mer genomic sequences, for a total probe of around 124-130-mers. DNA FISH/IF samples were imaged using a Leica SP8 upright Microscope, with a z-stack collected for each channel (step size, 0.15 or 0.3 um, frame interval 1 sec), using the oil objective HC PL APO CS2 63x/1.40. Images were processed by ImageJ (version 2.0.0/1.53c). The individual cells were identified by Hoechst/DAPI staining and cells containing signal for DNA-FISH channel were identified manually on the corresponding fluorescent channel. Distance between the DNA-FISH signal and the nucleolar marker immunofluorescence signal was calculated using ImageJ (version 2.0.0/1.53c) and used to count the number of foci contacting nucleolus and the number of cells with at least one contact with the nucleolus.

For manuscripts utilizing custom algorithms or software that are central to the research but not yet described in published literature, software must be made available to editors and reviewers. We strongly encourage code deposition in a community repository (e.g. GitHub). See the Nature Research guidelines for submitting code & software for further information.

Data

Policy information about availability of data

All manuscripts must include a data availability statement. This statement should provide the following information, where applicable:

- Accession codes, unique identifiers, or web links for publicly available datasets
- A list of figures that have associated raw data
- A description of any restrictions on data availability

All data supporting the findings of this study are available within the article and Supplementary Information, or from the corresponding author upon request.
All Nucleolar-DamIDseq, ChIPseq, HiC-rDNA and RNAseq data generated in this study are deposited in the Gene Expression Omnibus (GEO) database under the accession number GSE150822 (https://www.ncbi.nlm.nih.gov/geo/query/acc.cgi?acc=GSE150822).
The following public sequencing datasets were used in this study:
Replication timing ESCs (GEO: GSE95091, PMID: 29599440), A and B compartment (HiC ESCs) (GEO: GSE112222, PMID: 33433018), LADs in ESCs (GEO: GSE17051, PMID: 20513434), LADs in NPCs (GEO: GSE17051, PMID: 20513434), H3K4me3 ChIP in ESC (GEO: GSE23943, PMID: 22541430), H3K4me1 ChIP in ESC (GEO: GSE72164, PMID: 26637943), H3K27ac ChIP in ESC (GEO: GSE72164, PMID: 26637943), H3K27me3 ChIP in ESC (GEO: GSE23943, PMID: 22541430), H3K9me2 ChIP in ESC (GEO: GSE77420, PMID: 27315559), H3K9me3 ChIP in ESC (GEO: GSE23943, PMID: 22541430), CTCF ChIP in ESCs (GEO: GSE68582, PMID: 27152443), EZH2 ChIP in ESCs (GEO: GSE23943, PMID: 22541430), Ring1b ChIP in ESCs (GEO: GSE72164, PMID: 26637943), HiC ESCs (GEO: GSE96107, PMID: 29053968), HiC NPCs (GEO: GSE96107, PMID: 29053968), A and B compartment (HiC ESCs serum) (GEO: GSE96107, PMID: 29053968), A and B compartment (HiC NPCs) (GEO: GSE96107, PMID: 29053968), H3K4me3 ChIP in NPC (GEO: GSE96107, PMID: 29053968), H3K27ac ChIP in NPC (GEO: GSE96107, PMID: 29053968), H3K27me3 ChIP in NPC (GEO: GSE96107, PMID: 29053968), H3K9me3 ChIP in NPC (GEO: GSE96107, PMID: 29053968), H3K9me2 ChIP in NPC (GEO: GSE122263, PMID: 31488723), NADs in ESCs (4D Nucleome data portal 4DNESXE9K9DB, PMID: 32219510), NADs in ESCs (GEO: GSE103610, PMID: 32160538), NADs in MEFs (4D Nucleome data portal 4DNES15QV1OO, PMID: 31201210).

Field-specific reporting

Please select the one below that is the best fit for your research. If you are not sure, read the appropriate sections before making your selection.

☒ Life sciences ☐ Behavioural & social sciences ☐ Ecological, evolutionary & environmental sciences

For a reference copy of the document with all sections, see nature.com/documents/nr-reporting-summary-flat.pdf

Life sciences study design

All studies must disclose on these points even when the disclosure is negative.

Sample size	A minimum of 3 replicates was used for the Reverse Transcriptase-quantitative PCR . The choice of 3 replicates is commonly used by studies in the field. A minimum of 2 replicates was used for all of the sequencing-related experiments (Nucleolar-DamID, ChIP-Seq, RNA-Seq). The choice of 2 replicates is commonly used by studies in the field.
Data exclusions	No data have been excluded from the analysis
Replication	Reproducibility of experimental findings was assessed with replicates and other independent experiments. Nucleolar-DamID experiments, ChIPseq have been performed in duplicates for each condition discussed in the paper, while RNAseq was performed in triplicates. All

	replicates performed were successful and included in the manuscript and database on GEO https://www.ncbi.nlm.nih.gov/geo/query/acc.cgi?acc=GSE150822.
Randomization	No randomization has been performed for this study.
Blinding	Blinding was not possible here because we performed experimental treatments, and one investigator prepared, collected and analyzed the experimental treatments.

Reporting for specific materials, systems and methods

We require information from authors about some types of materials, experimental systems and methods used in many studies. Here, indicate whether each material, system or method listed is relevant to your study. If you are not sure if a list item applies to your research, read the appropriate section before selecting a response.

Materials & experimental systems

n/a	Involved in the study
☐	☒ Antibodies
☐	☒ Eukaryotic cell lines
☒	☐ Palaeontology and archaeology
☒	☐ Animals and other organisms
☒	☐ Human research participants
☒	☐ Clinical data
☒	☐ Dual use research of concern

Methods

n/a	Involved in the study
☐	☒ ChIP-seq
☒	☐ Flow cytometry
☒	☐ MRI-based neuroimaging

Antibodies

Antibodies used	Antibodies used in this study are listed and described in Supplementary Table 12.<br>Ab FISH: Nucleolin (rabbit polyclonal, Abcam, Cat# ab22758)<br>Ab IF: NPM1 (mouse monoclonal, Sigma, Cat# B0556), Fibrillarin (rabbit monoclonal, CellSignaling, Cat# 2639)<br>Secondary antibodies: IgG (H+L) Highly Cross-Adsorbed Alexa Fluor 488 (rabbit polyclonal, ThermoFischer, Cat# A11034), IgG (H+L) Highly Cross-Adsorbed Alexa Fluor 546 (rabbit polyclonal, ThermoFischer, Cat# A11035)<br>Ab ChIPseq: H3K9me2 (mouse monoclonal, Abcam, Cat# ab1220, lot:GR183500-3), H3K9me3 (rabbit polyclonal, Abcam, Cat# ab8898, lot:GR3217826-1).
Validation	All antibodies used in this study are commercially available. Validation was assessed by the company (see corresponding manufacture's web site).<br>Ab FISH: Nucleolin (https://www.abcam.com/nucleolin-antibody-ab22758.html)<br>Ab IF: NPM1 (https://www.sigmaaldrich.com/CH/it/product/sigma/b0556), Fibrillarin (https://www.cellsignal.com/products/primary-antibodies/fibrillarin-c13c3-rabbit-mab/2639)<br>Secondary antibodies: IgG (H+L) Highly Cross-Adsorbed Alexa Fluor 488 (https://www.thermofisher.com/antibody/product/Goat-anti-Rabbit-IgG-H-L-Highly-Cross-Adsorbed-Secondary-Antibody-Polyclonal/A-11034), IgG (H+L) Highly Cross-Adsorbed Alexa Fluor 546 (https://www.thermofisher.com/antibody/product/Goat-anti-Rabbit-IgG-H-L-Highly-Cross-Adsorbed-Secondary-Antibody-Polyclonal/A-11035)<br>Ab ChIPseq: H3K9me2 (https://www.abcam.com/histone-h3-di-methyl-k9-antibody-mabcam-1220-chip-grade-ab1220.html), H3K9me3 (https://www.abcam.com/histone-h3-tri-methyl-k9-antibody-chip-grade-ab8898.html).

Eukaryotic cell lines

Policy information about cell lines

Cell line source(s)	Mouse embryonic stem cells and the cell lines generated for this study were derived from E14 129/Ola. HEK 293T cells were derived from ATCC.
Authentication	All cell lines used in this study were genotyped and their identity authenticated by several means including resistance to drug selection and RNAseq.
Mycoplasma contamination	Tests for mycoplasma contamination are routinely performed (one a month). All cell lines used in this study are mycoplasma free
Commonly misidentified lines<br>(See ICLAC register)	No cell lines used in this study were found in the database of commonly misidentified cell lines that is maintained by ICLAC and NCBI Biosample.

ChIP-seq

Data deposition

☒ Confirm that both raw and final processed data have been deposited in a public database such as GEO.

☒ Confirm that you have deposited or provided access to graph files (e.g. BED files) for the called peaks.

Data access links<br>*May remain private before publication.*	https://www.ncbi.nlm.nih.gov/geo/query/acc.cgi?acc=GSE150822
Files in database submission	GSM4559238 ESC H2B-Dam repl1<br>GSM4559239 ESC H2B-Dam repl2<br>GSM4559240 ESC H2B-Dam-NoLS repl1<br>GSM4559241 ESC H2B-Dam-NoLS repl2<br>GSM4559242 ESC input<br>GSM4559243 ESC +RNA Ctrl -H3K9me2<br>GSM4559244 ESC +pRNA -H3K9me2<br>GSM4559245 ESC +RNA Ctrl -H3K9me3<br>GSM4559246 ESC +pRNA -H3K9me3<br>GSM4559247 ESC RNA-seq repl1<br>GSM4559248 ESC RNA-seq repl2<br>GSM4559249 ESC RNA-seq repl3<br>GSM5221275 NPC H2B-Dam repl1<br>GSM5221276 NPC H2B-Dam repl2<br>GSM5221277 NPC H2B-Dam-NoLS repl1<br>GSM5221278 NPC H2B-Dam-NoLS repl2<br>GSM5221279 ESC input repl2<br>GSM5221280 ESC +RNA Ctrl -H3K9me2 repl2<br>GSM5221281 ESC +pRNA -H3K9me2 repl2<br>GSM5221282 ESC +RNA Ctrl -H3K9me3 repl2<br>GSM5221283 ESC +pRNA -H3K9me3 repl2<br>GSM5221284 NPC repl1<br>GSM5221285 NPC repl2<br>GSM5221286 NPC repl3
Genome browser session<br>(e.g. UCSC)	No longer applicable.

Methodology

Replicates	Number of replicates has been specified in the manuscript.
Sequencing depth	All information for sequencing depth can be found in M&M. Nucleolar-DamID samples have a sequencing depth between 30 and 50 reads, ChIPseq between 15 and 30 mio reads, and RNAseq between 30 and 50 mio reads.
Antibodies	All information concerning the antibodies used for ChIPseq were described in details in Suppl. Table 12. Ab ChIPseq: H3K9me2 (mouse monoclonal, Abcam, Cat# ab1220, lot:GR183500-3), H3K9me3 (rabbit polyclonal, Abcam, Cat# ab8898, lot:GR3217826-1).
Peak calling parameters	For ChIPseq no peak calling was performed, but read coverage for 20kb bin region of H3K9me2 and H3K9me3 ChIPseq, "multiBamSummary" from deepTools (version 3.2.1) was calculated in order to identify regions with differential levels of the H3K9me2 and H3K9me3 upon addition of pRNA to the ESCs.
Data quality	Data quality has been described in details in M&M section
Software	All software used for analysis have been described in M&M, including the corresponding references
