# Supplementary figures and images for: Genome-wide maps of nucleolus interactions reveal distinct layers of repressive chromatin domains
Source: Nat Commun. 2022 Mar 18;13:1483. doi: 10.1038/s41467-022-29146-2 (PMC8933459; doi:10.1038/s41467-022-29146-2)

# Original blots and gels

Figure 1d

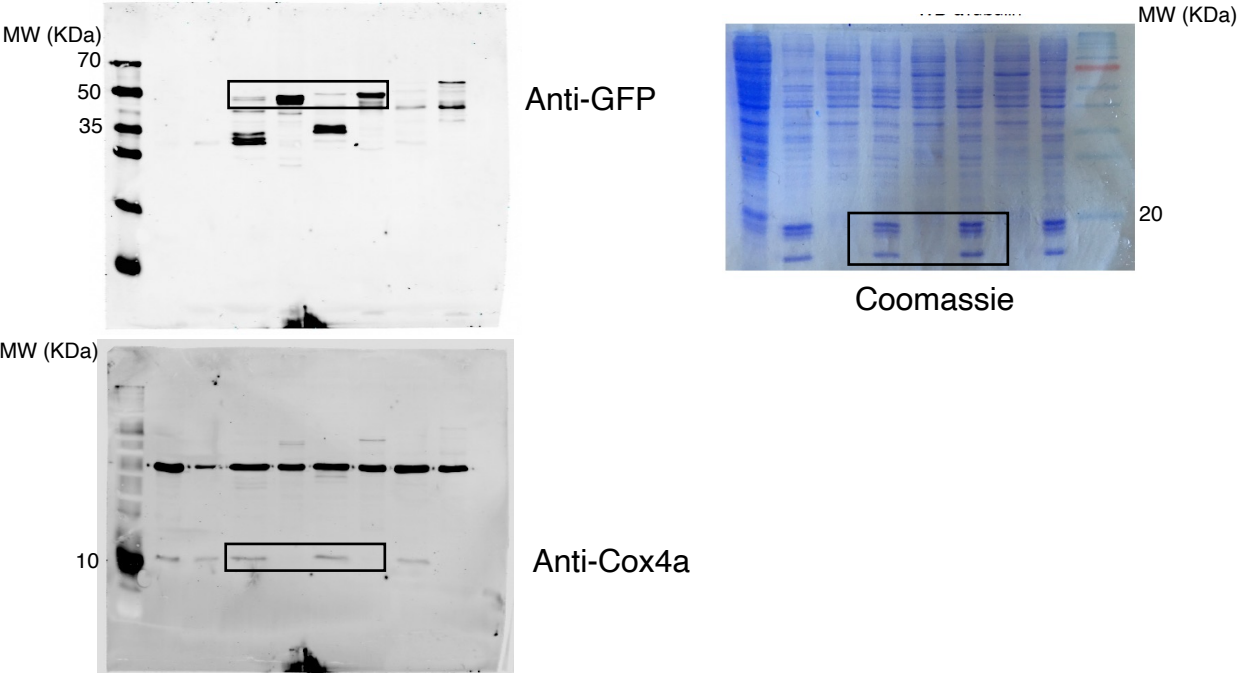

Suppl. Fig. 1a

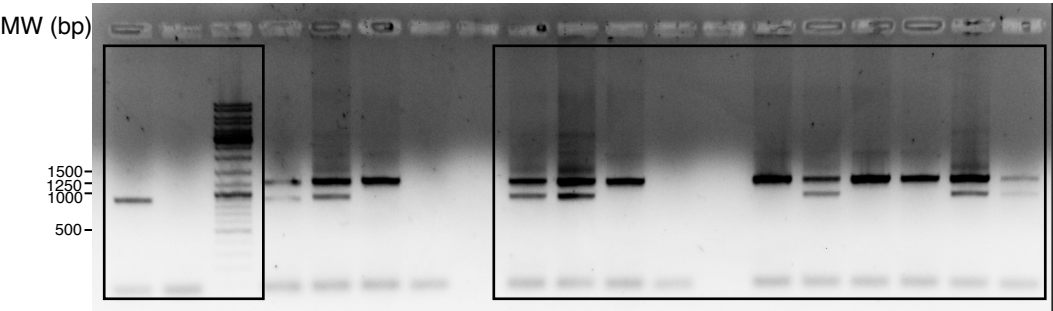

Supplement: Supplementary file 16 — Source data [file 41467_2022_29146_MOESM16_ESM.zip › Source data/Original_blot.pdf]
